# Systematic Analysis of Accuracy in Predicting Complete Oncological Resection in Pancreatic Cancer Patients—Proposal of a New Simplified Borderline Resectability Definition

**DOI:** 10.3390/cancers12040882

**Published:** 2020-04-04

**Authors:** Louisa Bolm, Katharina Mueller, Katharina May, Stefan Sondermann, Ekaterina Petrova, Hryhoriy Lapshyn, Kim Christin Honselmann, Dirk Bausch, Sergii Zemskov, Peter Bronsert, Tobias Keck, Steffen Deichmann, Ulrich F. Wellner

**Affiliations:** 1 Department of Surgery, University Medical Center Schleswig-Holstein, Campus Luebeck, 23562 Luebeck, Germany; louisa.bolm@googlemail.com (L.B.); katharina.mueller@uksh.de (K.M.); ekaterina.petrova@uksh.de (E.P.); hryhoriy.lapshyn@uksh.de (H.L.); kim.honselmann@uksh.de (K.C.H.); steffen.deichmann@uksh.de (S.D.); ulrich.wellner@uksh.de (U.F.W.); 2 Department of Radiology, University Medical Center Schleswig-Holstein, Campus Luebeck, 23562 Luebeck, Germany; katharina.may@uksh.de; 3 Department of Neuroradiology, University Medical Center Schleswig-Holstein, Campus Luebeck, 23562 Luebeck, Germany; 4 Department of Surgery, Marien Hospital, University Hospital of Ruhr University Bochum, 44621 Herne, Germany; dirk.bausch@dbausch.de; 5 Department of General Surgery, Bogomolets National Medical Unoversity, 01601 Kiev, Ukraine; szemskovmeister@gmail.com; 6 Department of Pathology, Medical Center—University of Freiburg, Faculty of Medicine, University of Freiburg, 79106 Freiburg, Germany; peter.bronsert@uniklinik-freiburg.de; 7 German Cancer Consortium (DKTK), Partner Site Freiburg, 79106 Freiburg, Germany

**Keywords:** pancreatic cancer, oncological resection, borderline resectability

## Abstract

*Background:* Borderline resectability in pancreatic cancer (PDAC) is currently debated. *Methods:* Patients undergoing pancreatic resections for PDAC were identified from a prospectively maintained database. As new borderline criteria, the presence of any superior mesenterico-portal vein alteration (SMPV) and perivascular stranding of the superior mesenteric artery (SMA) was evaluated in preoperative imaging. The accuracy of established radiological borderline criteria as compared to the new borderline criteria in predicting R status (sensitivity/negative predictive value) and overall survival was assessed. *Results:* 118 patients undergoing pancreatic resections for PDAC from 2013 to 2018 were identified. Forty-three (36.4%) had radiological perivascular SMA stranding and 55 (46.6%) had SMPV alterations. Interrater reliability was 90% for SMA stranding and 87% for SMPV alterations. The new borderline definition including SMPV alterations and perivascular SMA stranding was the best predictor of conventional R status (*p* = 0.040, sensitivity 53%, negative predictive value 81%) and Leeds/Wittekind circumferential margin status (*p* = 0.050, sensitivity 73%, negative predictive value 79%) as compared to established borderline resectability definition criteria. Perivascular SMA stranding qualified as an independent negative prognostic parameter (HR 3.066, 95% CI 1.078–5.716, *p* = 0.036). *Conclusion*: The radiological evaluation of any SMPV alteration and perivascular SMA stranding predicts R status and overall survival in PDAC patients, and may serve to identify potential candidates for neoadjuvant therapy.

## 1. Introduction

Pancreatic ductal adenocarcinoma (PDAC) is an aggressive solid malignancy associated with poor prognosis [1,2]. The majority of patients present with distant metastases at the time of diagnosis, and less than 50% of all PDAC patients are eligible for upfront curative resection [3].

Over the past years, attempts were made to specify resectability criteria in pancreatic cancer patients. While irresectability is well-defined, the definition of borderline resectability is currently debated. Borderline resectable PDAC tumors are technically resectable at a high risk of margin positive resection [4]. In 2014, the International Study Group of Pancreatic Surgery (ISGPS) aimed to standardize the definition and approach to borderline resectable PDAC [5]. The criteria of borderline resectability were adopted from the National Comprehensive Cancer Network (NCCN) definition, comprising the absence of distant metastases, the involvement of the superior mesenterico-portal vein (SMPV) and tumor abutment of a maximum of 180° in the superior mesenteric artery (SMA). The 2014 ISGPS definition relies on anatomic criteria evaluated preoperatively. As a major obstacle, these criteria cannot always be safely determined in preoperative imaging. Therefore, intraoperative re-evaluation is necessary and may often reveal criteria of irresectability.

In 2017, the International Association of Pancreatology (IAP) added new dimensions to the definition of borderline resectability [6]. In addition to anatomical criteria, aspects of tumor biology in terms of elevated serum CA19-9 levels and patient performance status were defined as further determinants of borderline resectability. Anatomical criteria mainly regarding the involvement of the SMPV were refined by further amendments. By adding these additional aspects, the definition of borderline resectability has become increasingly complex, and translation to clinical practice remains challenging.

The main aim in borderline resectable PDAC is to safely achieve margin negative resection. Despite updated borderline resectability definitions, preoperative parameters predicting R0 resections in borderline resectable PDAC patients are lacking. The accuracy of R status prediction by distinct borderline resectability definitions has not been assessed systematically yet. Mainly in the light of neoadjuvant therapy, the preoperative imaging-based prediction of R status is of major importance. Those PDAC patients with a high risk of margin positive resections should be considered for neoadjuvant therapy. 

The accuracy of established borderline criteria in predicting R status is unclear, and their applicability to clinical routine appears limited due to complex scoring systems. We intended to develop simplified preoperative radiological criteria safely predicting R status. The aim of this study was to systematically assess the prediction of R status, comparing established borderline resectability criteria and novel radiological parameters by the means of a diagnostic accuracy study. Our hypothesis is that a simplified borderline resectability scoring system would improve both the quality of R status prediction and the willingness of radiologists and surgeons to integrate the scoring system into clinical routine.

## 2. Results

### 2.1. Baseline Parameters and Perivascular SMA Stranding

A total of 118 patients undergoing pancreatoduodenectomy (PD) for PDAC in the period from 2013 to 2018 were identified, and the median follow-up was 12 months. The median age at operation was 68 years, ranging from 41 to 85. The median preoperative serum CA19-9 level was 121 IU/mL, ranging from 0.06 to 2945 IU/mL. According to the ISGPS 2014 definition, 50 patients (42.4%) were classified as borderline resectable. According to the IAP 2017 definition, 88 patients (74.6%) were considered borderline resectable. Sixty-one patients (51.7%) underwent a pylorus-preserving pancreatoduodenectomy (PPPD), 16 patients (13.6%) had a Whipple procedure, 19 patients (16.1%) received a distal pancreatectomy and 22 patients (18.6%) had a total pancreatectomy. A total of 38 (32.2%) patients underwent portal vein resections (PVRs).

In a pilot study, the interrater reliability for the novel borderline criteria (SMA stranding and SMPV alterations) was assessed for two surgeons and two radiologists blinded for outcome variables. The raters evaluated SMA stranding and SMPV alterations as either positive or negative in 30 PDAC patients based on preoperative CT-based imaging. The interrater reliability was 90% (Kappa = 0.77) for SMA stranding and 87% (Kappa 0.75) for SMPV alterations. 

For the study cohort of 118 patients, the novel borderline resectability criteria were assessed by an independent radiologist and two independent trained surgeons blinded for patient outcome. A total of 43 patients (36.4%) had positive perivascular SMA stranding. Fifty-three patients (54.9%) had at least one SMPV alteration. Patients with SMA stranding were more often diagnosed as borderline resectable according to the ISGPS 2014 criteria (30.7% vs. 62.8%, *p* = 0.001) and according to the IAP 2017 definition (65.3% vs. 90.7%, *p* = 0.002). Furthermore, SMPV alterations were more frequent in patients with SMA stranding (67.4% vs. 32.0%, *p* = 0.001). Positive N status (81.4% vs. 61.3%, *p* = 0.025) and perineural invasion (90.5% vs. 73.3%, *p* = 0.032) were more often detected in patients with SMA stranding. Differences in baseline parameters between patients with SMA stranding vs. no stranding are displayed in Table 1.

### 2.2. Borderline Resectability Definitions and Accuracy in the Prediction of R Status

Sensitivity of borderline resectability definition criteria in predicting positive R status was calculated. Furthermore, the negative predictive value predicting R0 resection in the absence of positive borderline criteria was calculated. The ISGPS 2014 definition had a low sensitivity and low negative predictive value (NPV) in predicting UICC/AJCC (= conventional) R status (sensitivity 48%, NPV 64%) and Leeds/Wittekind R status (sensitivity 70% and NPV 60%). The IAP 2017 definition also had a low sensitivity and NPV regarding the prediction of conventional R status (sensitivity 43%, NPV 31%) and Leeds/Wittekind R status (sensitivity 69% and NPV 29%). SMPV alterations were associated with an improved sensitivity and NPV in predicting conventional R status (sensitivity 45%, NPV 60%) and Leeds/Wittekind R status (sensitivity 70% and NPV 60%). Perivascular SMA stranding was associated with a high sensitivity and NPV in predicting conventional R status (sensitivity 53%, NPV 73%) and Leeds/Wittekind R status (sensitivity 80% and NPV 76%). The combination of SMPV alterations and SMA stranding as novel borderline resectability definition criteria was associated with an increased sensitivity and an even higher NPV in predicting conventional R status (sensitivity 53%, NPV 80%) and Leeds/Wittekind R status (sensitivity 73% and NPV 79%). Further details are displayed in Table 2. Regarding conventional R status, patients undergoing PVRs were not more likely to undergo R1 resection (SMV resection: 47.4% R1, versus no SMV resection: 33.8% R1; *p* = 0.0163). However, patients with PVRs more often showed R1 resection according to Leeds/Wittekind R status (SMV resection: 81.6% R1 versus no SMV resection: 61.3% R1).

**Figure 1:**
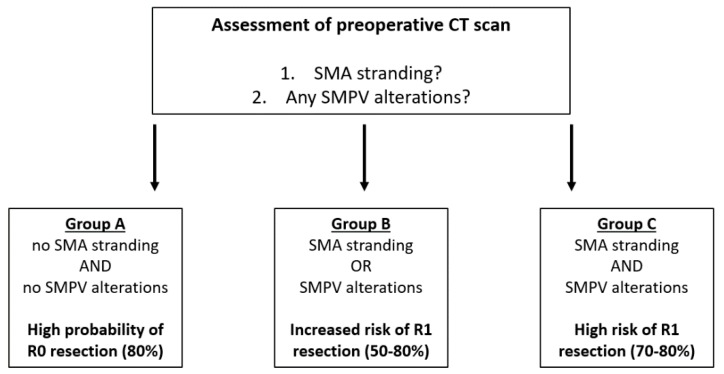
A novel scoring system for borderline resectability. SMA: superior mesenteric artery; SMPV: superior mesenterico-portal vein.

Based on the high accuracy of the novel radiological parameters in predicting R status, a new borderline resectability scoring system was developed. This scoring system divides patients into three major subgroups based on the probability of R1 or R0 resection. Group A is defined as patients with a high probability of R0 resection (80%); these patients show neither SMA stranding nor SMPV alterations. Patients in group B have an increased risk of R1 resection (50%–80%) and present with either SMA stranding or SMPV alterations in preoperative imaging. Group C is defined as patients with a high risk of R1 resection (70%–80%), showing both SMA stranding and SMPV alterations. Figure 1 displays an overview of the groups.

### 2.3. Survival Analysis Total Cohort

**Figure 2:**
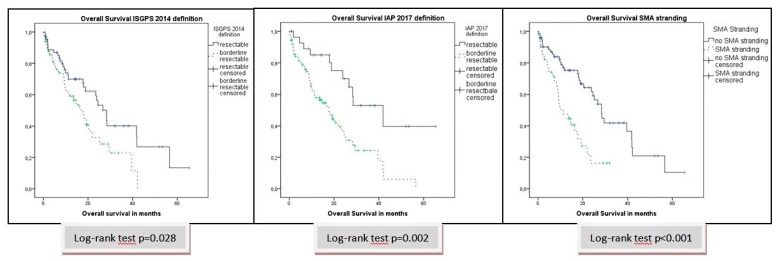
Borderline resectability definitions and overall survival. SMA: superior mesenteric artery; ISGPS: International Study Group of Pancreatic Surgery; IAP: International Association of Pancreatology.

Median overall survival (OS) in all patients was 12 months. Prognostic parameters in the univariate analysis were the ASA score (ASA 0–2: 31 months vs. 3–4: 22 months, *p* = 0.044), the ISGPS 2014 borderline criteria (borderline: 19 months vs. resectable: 30 months, *p* = 0.030), the IAP 2017 borderline criteria (borderline: 20 months vs. resectable: 40 months, *p* = 0.002), SMPV alterations (SMPV alterations: 18 months vs. no SMPV alterations: 31 months, *p* = 0.017), SMA stranding (positive stranding: 13 months vs. no stranding: 30 months, *p* < 0.001), the novel borderline definition (borderline: 13 months vs. resectable: 29 months, *p* = 0.006), the operation time (>374 min: 18 months vs. <374 min: 30 months, *p* = 0.003), portal vein resection (PVR 18 months: vs. no PVR: 31 months, *p* = 0.012), N stage (N0: 33 months vs. N+: 20 months, *p* = 0.022), lymphovascular invasion (L+: 17 months vs. L0: 31 months, *p* = 0.002), vascular invasion (V+: 14 months vs. V0: 30 months, *p* = 0.001), perineural invasion (Pn+: 22 months vs. Pn0: 40 months, *p* = 0.023), UICC/AJCC R status (R0: 30 months, vs. R+: 18 months, *p* = 0.016) and Leeds/Wittekind R status (R0 wide: 34 months vs. R0 narrow/R1: 21 months, *p* = 0.041). For the survival curves of negative versus positive SMA stranding, see Figure 2.

In the multivariate analysis, SMA stranding (HR 3.066, 95% CI 1.078–5.716, *p* = 0.036), operation time (HR 1.989, 95% CI 1.055–3.748, *p* = 0.044) and vascular invasion (HR 2.375, 95% CI 1.201–4.700, *p* = 0.013) remained independent prognostic parameters. Details of the survival analysis are displayed in Supplementary Table S1.

## 3. Discussion

In the current study, a new simplified borderline resectability scoring system was developed based on perivascular stranding of the superior mesenteric artery (SMA) and any alterations of the superior mesenterico-portal vein (SMPV). The scoring system identifies patients with a high probability of R0 resection (group A), patients with an increased risk of R1 resections (group B) and patients with a high risk of R1 resection (group C). The current study was the first, to the best of our knowledge, to systematically evaluate the accuracy of borderline resectability definitions in predicting R status. The novel simplified borderline scoring system qualified best to predict conventional R status by UICC/AJCC R status and Leeds/Wittekind circumferential margin status—better than the ISGPS 2014 and IAP 2017 borderline resectability definitions. Perivascular SMA stranding is associated with perineural invasion and remained an independent prognostic parameter with a negative impact on long-term overall survival. Thus, we developed a scoring system simplifying the diagnostic process for borderline resectable PDAC patients and developed a tool to accurately predict R status and patient prognosis. 

Borderline resectable PDAC is considered resectable with a high risk of macroscopic or microscopic margin positive resection [4]. In 2014, a consensus statement by the international study group of pancreatic surgery (ISGPS) aimed to precisely separate borderline PDAC from irresectable PDAC, and to standardize the definition criteria based on preoperative radiological parameters [5]. While any SMPV occlusion and any arterial encasement had been considered a criterion of irresectability before, the ISGPS followed the National Comprehensive Cancer Network (NCCN) definition of borderline resectability and classified SMPV distortion and even short-segment venous occlusion—as well as the encasement of the gastroduodenal or hepatic artery and up to 180° abutment of the SMA—as borderline resectable cases. Meta-analyses and large national cohort studies had shown higher rates of postoperative morbidity, but comparable rates of postoperative mortality and long-term overall survival after PVRs, as compared to patients without venous resections for PDAC [6,7]. In consequence, PVR was no longer considered an obstacle for curative resection if venous reconstruction was feasible. Arterial resections, mainly resections of the SMA, are still not routinely performed because of excessive morbidity and mortality rates in these patients [8]. No patient in the study cohort underwent SMA resection.

The ISGPS and NCCN [8] borderline criteria from 2014 were adopted by the current German national S3-guidelines setting the standards of pancreatic cancer treatment in Germany [9]. Consequently, German centers of pancreatic surgery are obligated to apply these mainly anatomical criteria to identify patients with borderline resectable pancreatic cancer. 

As a major obstacle, the preoperative CT-based assessment of venous and arterial involvement may be inaccurate in a considerable number of patients; the sensitivity of arterial and venous invasion was demonstrated to be as low as 66% and 14% respectively [10]. Furthermore, the false positive assessment of arterial encasement occurs frequently [11]. Joo et al. recently performed a study assessing interrater reliability among radiologists evaluating resectability in PDAC [12]. The authors demonstrated an interrater reliability as low as 30%, and in cases determined as borderline resectable, resection rates varied from 0% to 74%. In consequence, the exploration of patients diagnosed as borderline resectable is currently necessary to verify inconsistent resectability determinations based on preoperative imaging. 

In our study, the sensitivities of the ISGPS 2014 definition in predicting R status, both the UICC/AJCC R status and Leeds/Wittekind circumferential margin status, were as low as 48% and 60%, respectively. Furthermore, the accuracy in predicting margin negative resection in the absence of positive ISGPS 2014 borderline resectability criteria was low, at 64% and 60%, respectively.

In 2017, the International Association of Pancreatology (IAP) aimed to improve the accuracy of the borderline resectability definition by adding further biological and conditional dimensions [13]. Several studies demonstrated reduced resection rates and impaired overall survival after resection for patients with high preoperative carbohydrate antigen 19-9 (CA19-9) levels and preoperative suspicions of lymph node metastases [14,15,16]. The IAP 2017 borderline resectability definition introduced preoperative CA19-9 levels of more than 500 IU/mL and imaging-based suspicions of lymph node metastases as novel borderline resectability criteria. Additionally, based on a study identifying performance status as a major prognostic parameter in PDAC, a performance status of more than 2 was added as a conditional criterion of borderline resectability [17]. By expanding the definition of borderline resectability, decision making to determine resectability becomes more and more complex. In consequence, a general use of the IAP 2017 borderline definition could not be imposed internationally.

Kato et al. compared the ISGPS 2014 and NCCN guidelines to the IAP 2017 borderline definition [18]. The study disclosed that the IAP 2017 definition allowed the accurate stratification of resectable, borderline resectable and unresectable pancreatic cancers, with significantly different long-term survival estimates. In contrast, the ISGPS/NCCN-based stratification of resectability could not distinguish between different prognostic subgroups. In the current study, both the ISGPS 2014 and IAP 2017 borderline criteria qualified as prognostic parameters in the univariate analysis. However, in the multivariate analysis, only SMA stranding, but not the other borderline criteria, remained an independent prognostic factor.

In the current study, perivascular SMA stranding was evaluated as potential borderline resectability criterion. Perivascular SMA stranding in PDAC patients can be visualized in preoperative CT-based imaging. In previous studies, this phenomenon was described as cancer-infiltration-mimicking fibrous adhesion or an inflammatory reaction [5,10,11]. Kato et al. found impaired overall survival rates and a higher rate of early distant metastasis in patients with a reticular pattern surrounding the SMA [19]. We recently demonstrated that perivascular SMA stranding is a preoperative radiological marker of fibrotic stroma resection (S+ status) at the mesopancreatic resection margin [20]. In this study, S+ status was a strong negative prognostic parameter and served as a surrogate of aggressive tumor biology and advanced tumor cell dissemination. These results are in line with the current study. SMA stranding was associated with perineural invasion and lymph node infiltration, mirroring increased local tumor invasion. It may also be speculated that perivascular SMA stranding is a morphological correlate of very pronounced perineural invasion. Perineural invasion is well-known as one of the main causes of local tumor recurrence and metastasis and was demonstrated as one of the major prognostic parameters in PDAC [21,22,23]. In the current study, perivascular SMA stranding qualified as an independent prognostic factor, stronger than established prognostic parameters like ASA score, N status, lymphovascular invasion or R status.

Alterations of the SMPV were assessed as a further criterion of borderline resectability in the current study. The prognostic relevance of the amount of SMPV tumor involvement, tumor localization, amount of encasement, distortion or occlusion is under debate, and recent studies present conflicting results [4,13,24,25]. The most probable cause of these heterogenous results is the difficulty in assessing the SMPV circumferentially in preoperative imaging. To simplify the evaluation of preoperative imaging and to improve reproducibility, we decided to assess any alterations of SMPV—such as tumor narrowing, distortion or occlusion—as a borderline resectability criterion. A current study demonstrated that any irregularity of the SMPV contour is predictive of venous involvement and also qualifies as a negative prognostic factor [26]. In our study, SMPV alterations qualified as a negative predictive parameter in the univariate analysis, but not in the multivariate analysis. The criterion of SMPV was most likely ruled out by other strong independent prognostic factors, such as SMA stranding or N status. However, SMPV alterations are an important component of the novel borderline resectability definition, as they considerably increase the accuracy of R status prediction as compared to SMA stranding alone. Combining both SMA stranding and SMPV alterations, the novel borderline resectability scoring system qualifies to safely predict R0 resection (group A), and to identify patients with an increased risk of R1 resection (group B) and patients with a high risk of R1 resection (group C) based on simple preoperative radiological criteria.

R0 resection is the major outcome parameter in borderline resectable patients, and R status is one of the most important prognostic parameters in pancreatic cancer patients [27,28,29]. However, the current study is the first, to our knowledge, evaluating the accuracy of different borderline definition criteria in predicting R status. In general, borderline resectable PDAC is defined as technically resectable with a high risk of margin positive resection. The sensitivity of ISGPS 2014 and IAP 2017 definitions in predicting R status rangedaround 50%, and thus did not qualify to predict a high risk (>50%) of margin positive resection. In contrast, the novel borderline criteria for SMA stranding and SMPV alterations together had a high sensitivity mainly in predicting Leeds/Wittekind R status, mirroring the actual definition of borderline resectability (increased risk of R1 resection). Furthermore, the probability of R0 resection in the absence of the novel borderline criteria was as high as 70% to 80%, as compared to 30% to 50% for the ISGPS 2014 and IAP 2017 definition criteria. Consequently, the novel borderline definition may serve as an important tool to safely achieve R0 resections.

As a major obstacle, the pathological work-up of resection margins is not standardized internationally. The UICC/AJCC criteria, mainly applied in the United States, consider resection margins positive only in case of tumor cells in direct contact with the resections margin. The Leeds/Wittekind protocol used in Europe involves a circumferential assessment of all tumor margins, and a distinction is made between “R0 narrow”, with less than 1 mm distance between tumor cells and the resection margin, and “R0 wide”, showing a minimum distance of 1 mm between tumor cells and the resection margin [30,31]. Several studies and meta-analyses have demonstrated improved overall survival rates following R0 wide resections as compared to R0 narrow and R1 resections [27,32,33]. The novel borderline definition criteria show a moderate sensitivity in predicting UICC/AJCC R status and a high sensitivity in predicting Leeds/Wittekind circumferential margin status.

This study is limited by its retrospective nature, and potential bias or cofounders cannot be completely ruled out. To validate the results of this study, future large-scale cohort studies or prospective trials should be performed.

Mainly in borderline resectable PDAC, neoadjuvant therapy is of growing importance. Patients in the current study were not treated with neoadjuvant therapy. The German S3-guideline for the treatment of pancreatic cancer recommends upfront resection in borderline resectable PDAC patients, and neoadjuvant therapy is only allowed in the context of clinical studies [9]. However, current studies show promising results for neoadjuvant treatment in borderline resectable PDAC patients [34,35]. Multiple studies and meta-analyses demonstrated a higher R0 resection rate and improved overall survival rates in borderline resectable PDAC patients undergoing neoadjuvant therapy [36,37,38]. Different treatment regimens are currently assessed in randomized trials for borderline resectable PDAC patients. The ALLIANCE A021101 pilot study recently demonstrated an overall survival of 22 months and an R0 resection rate as high as 64% in borderline resectable PDAC patients undergoing neoadjuvant therapy with modified FOLFIRINOX and stereotactic radiotherapy [39]. The benefit of radiotherapy, as compared to FOLFIRINOX-based chemotherapy alone, remains unclear and will be further evaluated in the ALLIANCE A021501 trial [40]. However, due to the various complex definitions of borderline resectability, patient selection for neoadjuvant therapy is challenging and has not yet been standardized. The novel borderline resectability scoring system may serve to simplify patient selection for neoadjuvant therapy. Mainly patients with a considerable risk of R1 resection should undergo neoadjuvant therapy. Based on the results of the current study, we propose to consider neoadjuvant therapy in group B patients with an increased risk of R1 resection and in group C patients with a high risk of R1 resection, so as to improve R0 resection rates and, potentially, overall survival rates as well. The benefit of neoadjuvant therapy in these patient subgroups should be assessed in future studies. Furthermore, potential molecular markers may improve the accuracy of the novel borderline resectability scoring system and should be addressed in the future.

## 4. Materials and Methods 

### 4.1. Patients and Study Parameters

Approval for the study was obtained from the University of Luebeck’s institutional ethics committee (#17-118A, 2017). Patients who underwent PD for PDAC were identified from a prospectively maintained database. No neoadjuvant therapy was performed. Patients with locally advanced PDAC tumors were excluded from the study. Patients included in the study underwent surgery in the period from 2013 to 2018. The following patient baseline parameters were included for the analysis: age, gender, American Society of Anesthesiologists score (ASA score) and preoperative serum levels of carbohydrate antigen 19-9 (CA19-9) in IU/mL. ASA score was dichotomized as ASA scores 0–2 versus 3–4, and serum CA19-9 was dichotomized according to the upper normal serum value of 40I U/mL. The operation parameters analyzed in the study were the type of resection (Whipple, pylorus-preserving PD (PPPD), total pancreatectomy (TPE) or distal pancreatectomy), segmental portal vein resection (PVR), multivisceral resection and operation time (from incision to skin closure). Operation time was dichtomized according to the median.

Preoperative CT-based imaging was reviewed by one radiologist and two trained surgeons for the evaluation of borderline resectability criteria.

### 4.2. Tests of the Diagnostic Accuracy of the Borderline Resectability Criteria

**Figure 3:**
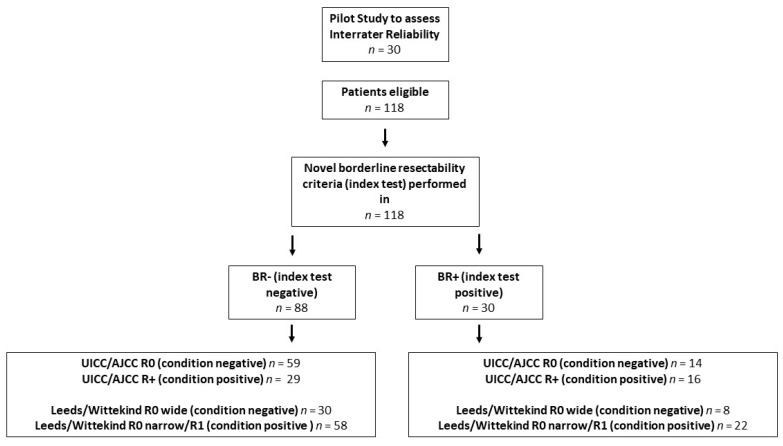
A Study Flow Chart according to the STARD 2015 List of essential items for reporting diagnostic accuracy studies. BR-: not borderline resectable; BR+: borderline resectable; UICC/AJCC: Union International Contre le Cancer/American Joint Committee on Cancer.

Accuracy assessment of the borderline resectability criteria was performed following the STARD 2015 (a list of essential items for reporting diagnostic accuracy studies) [41]. For the study flow chart, see Figure 3; for list of STARD items, see Supplementary Table S1. CT scans and patients’ records were assessed for borderline resectability according to the ISGPS 2014 definitions [5], and according to the IAP 2017 definitions [13], as either resectable or borderline resectable. Both definitions served as established reference standard tests, as the internationally established and most frequently used borderline definition criteria. According to the ISGPS 2014 definition, borderline resectable pancreatic cancer is defined as [1] no evidence of distant metastases; [2] venous involvement of the superior mesenteric vein or portal vein, with distortion or narrowing of the vein or occlusion of the vein, with suitable vessels proximal and distal, allowing for safe resection and replacement; [3] gastroduodeneal artery encasement up to the hepatic artery, with either short segment encasement or direct or indirect abutment of the hepatic artery without extension to the celiac axis; and [4] tumor abutment of the SMA not exceeding 180° of the circumference of the vessel wall. According to the IAP 2017 definition, borderline resectable PDAC is defined as [1] a tumor contact angle of 180° or greater, or invasion of the SMV/PV with bilateral narrowing or occlusion, but not exceeding the inferior border of the duodenum; [2] tumor contact with the SMA or celiac axis at an angle of less than 180° without showing stenosis or deformity, or tumor abutment of the common hepatic artery without showing tumor contact with the proper hepatic artery and/or celiac axis; [3] clinical findings giving rise to the suspicion of distant metastases, including serum CA19-9 levels of more than 500 U/mL or regional lymph node metastasis; [4] patients with resectable PDAC, but a performance status of 2 or more [13].

**Figure 4:**
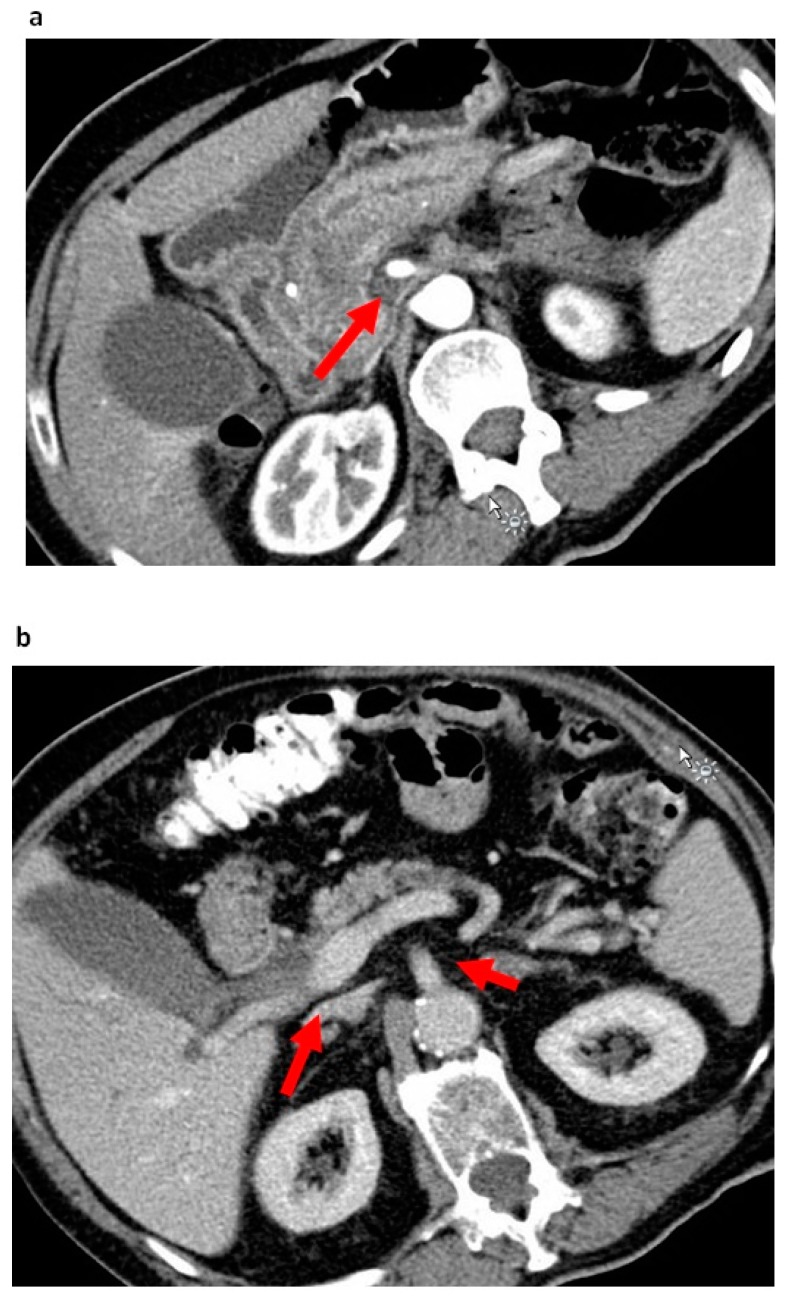
The novel resectability criteria: SMA stranding and superior mesenterico-portal vein alteration (SMPV) alterations. (**a**). Perivascular stranding of the superior mesenteric artery in a patient with pancreatic head cancer; (**b**) Tumor narrowing and distortion of the superior mesenteric vein and the absence of perivascular stranding of the superior mesenteric artery in a patient with pancreatic head cancer. SMA: superior mesenteric artery; SMPV: superior mesenterico-portal vein.

Furthermore, novel simplified borderline criteria were evaluated as an index test. Perivascular SMA stranding was considered positive in case of perivascular hazy density of the fat plane. Regarding the SMPV, any radiologically determinable vessel abnormalities such as tumor narrowing, vessel distortion or vessel occlusion were classified as SMPV alterations. The novel borderline resectability criteria are depicted in Figure 4a,b. In a pilot study of 30 patients, interrater reliability was assessed for the novel criteria. Two radiologists and two surgeons, blinded for clinical patient criteria and outcome variables, reviewed the CT-based imaging of 30 patients, assessing SMA stranding and SMPV alterations as either positive or negative. The interrater reliability for SMA stranding was 90%, while the interrater reliability for SMPV alterations was 87%. For the patients included in the study, preoperative CT-based imaging was reviewed by one independent radiologist and two independent surgeons blinded for clinical patient criteria and outcome variables. The evaluation of the ISGPS 2014 and IAP 2017 reference standard borderline resectability criteria—as well as of the novel radiological criteria, SMA alterations and SMPV alterations—was performed.

The histopathological parameters included in the analysis were the T stage, N stage and M stage grading according to Broders [42]; the lymph node ratio (LNR); lymphovascular invasion (L); vascular invasion (V) and perineural invasion (Pn). T stage was dichotomized as T1-2 versus T3-4, the LNR was dichotomized according to the median LNR value, and Broders grading was dichotomized as G1-2 versus G3-4. R status was re-evaluated by an experienced pathologist and two trained surgeons according to the UICC/AJCC criteria (conventional R status), as R0 if no tumor cells were detected at the resection margin, versus R1 if tumor cells were present right at the resection margin [43]. Specimens were further classified according to Leeds/Wittekind circumferential R status, as R1 if tumor cells were detected at the resection margin, versus R0 narrow if tumor cells were detected within 1 mm distance of the resection margin, versus R0 wide if the distance between tumor cells and resection margin was more than 1 mm [30,31]. TNM staging was performed according to the 7th edition of the American Joint Committee on Cancer (AJCC) [44]. The postoperative parameters were reoperation and adjuvant therapy. Overall survival time was defined from surgery until death from any cause.

For estimating the diagnostic accuracy of borderline resectability definitions in predicting R status, cross tabulation was performed to determine the sensitivity and negative predictive value of reference standards (ISGPS 2014 and IAP 2017) and the index test (SMA stranding and SMPV alterations). Sensitivity is defined as the proportion of patients with a positive test result (patients defined as borderline resectable) who have a positive condition (R+ resection). The negative predictive value is defined as the proportion of patients with a negative test result (patients defined as NOT being borderline resectable) who have a negative condition (R0 resection).

### 4.3. Statistics 

For statistical analysis, IBM SPSS Statistics for Windows, Version 25.0 was used. Continuous and categorical variables were expressed as median/range and absolute/relative frequencies, respectively. Statistical testing was performed by Chi-squared tests. Interrater reliability was calculated as the joint probability of agreement (percentage of the time the raters agree in the nominal rating system), and Fleiss’ Kappa was additionally calculated to assess interrater reliability for more than four raters. Median overall survival estimates were determined with the Kaplan–Meier method, the log-rank-test and the Cox proportional hazards model. Multivariable analysis was also performed with the Cox proportional hazards model. The significance level was set to *p* < 0.05 (two-sided). All confidence intervals (CI) reported are 95% confidence intervals.

## 5. Conclusions

In conclusion, the preoperative CT-based assessment of any SMPV alterations and of perivascular SMA stranding qualified as new definition criteria of borderline resectability in PDAC patients. These criteria may be applied to accurately predict R status. 

## Figures and Tables

**Table 1:** Baseline parameters in patients with superior mesenteric artery (SMA) stranding versus no SMA stranding.

Baseline Parameters SMA Stranding	No SMA Stranding	Positive SMA Stranding	
Total *n*	75	43	
**Parameter**	*n* (%)/median (range)	*n* (%)/median (range)	*p*-value
**Age**			
< 68	39 (52.0)	20 (46.5)	
> 68	36 (48.0)	23 (43.5)	0.702
**Sex**			
male	31 (41.3)	26 (60.5)	
female	44 (58.7)	17 (39.5)	0.060
**ASA Score**			
0–2	38 (50.7)	15 (34.9)	
3–4	37 (49.3)	28 (65.1)	0.125
**Serum CA 19-9**			
>40 U/L	22 (66.7)	20 (80.0)	0.375
**Borderline ISGPS 2014 Definition**			
BR-	52 (69.3)	16 (37.2)	
BR+	23 (30.7)	27 (62.8)	0.001
**Borderline IAP 2017 Definition**			
BR-	26 (34.7)	4 (9.3)	
BR+	49 (65.3)	39 (90.7)	0.002
**Any SMPV alterations**			
no	51 (68.0)	14 (32.6)	
yes	24 (32.0)	29 (67.4)	0.001
**PVR**			
no	50 (66.7)	30 (69.8)	
yes	25 (33.3)	13 (30.2)	0.839
**Multivisceral resection**			
no	70 (93.3)	38 (88.4)	
yes	5 (6.7)	5 (11.6)	0.494
**T stage**			
T1/2	17 (22.7)	10 (23.3)	
T3/4	58 (77.3)	33 (76.7)	1.000
**N stage**			
N0	29 (38.7)	8 (18.6)	
N+	46 (61.3)	35 (81.4)	0.025
**M**			
M0	72 (96.0)	41 (95.3)	
M1	3 (4.0)	2 (4.7)	1.000
**LNR**			
< median 0.08	43 (57.3)	17 (39.5)	
> median 0.08	32 (42.7)	26 (60.5)	0.085
**Grading**			
G 1/2	53 (71.6)	32 (84.2)	
G 3/4	21 (28.4)	6 (15.8)	0.167
**Lymphovascular invasion**			
L0	45 (60.0)	26 (60.5)	
L1	30 (40.0)	17 (39.5)	1.000
**Vascular invasion**			
V0	59 (78.7)	28 (65.1)	
V1	16 (21.3)	15 (34.9)	0.130
**Perineural invasion**			
Pn0	20 (26.7)	4 (9.5)	
Pn1	55 (73.3)	38 (90.5)	0.032
**Adjuvant therapy**			
no	35 (46.7)	26 (60.5)	
yes	40 (53.3)	17 (39.5)	0.182

SMA: superior mesenteric artery; BMI: body mass index; ASA score: American Society of Anesthesiologists score; CA19-9: carbohydrate antigen 19-9; ISGPS: International Study Group of Pancreatic Surgery; IAP: International Association of Pancreatology; SMPV: superior mesenterico-portal vein; PVR: portal vein resection; LNR: lymph node ratio.

**Table 2:** Borderline resectability and prediction of R status.

Borderline Resectability Definitions and Prediction of R Status								
	**UICC/AJCC R Status**							
		**R0**		**R+**				
		*n*/median	%/range	*n*/median	%/range	*p*-value	*Sensitivity*	*NPV*
total *n*		73	61.9	45	38.1			
**Borderline ISGPS 2014 Definition**	BR-	47	64.4	21	46.7	0.084	48%	64%
	BR+	26	35.6	24	53.3			
**Borderline IAP 2017 Definition**	BR-	23	31.5	7	15.6	0.081	43%	31%
	BR+	50	68.5	38	84.4			
**Any SMPV alterations**	no	44	60.3	21	46.7	0.183	45%	60%
	yes	29	39.7	24	53.3			
**SMA Stranding**	no	53	72.6	22	48.9	0.011	53%	73%
	yes	20	27.4	23	51.1			
**Borderline novel Definition**	BR-	59	80.8	29	64.4	0.040	53%	81%
	BR+	14	19.2	16	35.6			
		**Leeds/Wittekind R status**						
		**R0 wide**		**R0 narrow/R1**				
		*n*/median	%/range	*n*/median	%/range	*p*-value	*Sensitivity*	*NPV*
total *n*		38	32.2	80	67.8			
**Borderline ISGPS 2014 Definition**	BR-	23	60.5	45	56.3	0.695	60%	60%
	BR+	15	39.5	35	43.7			
**Borderline IAP 2017 Definition**	BR-	11	28.9	19	23.8	0.652	69%	29%
	BR+	27	71.1	61	76.2			
**Any SMPV alterations**	no	23	60.5	42	52.5	0.436	70%	60%
	yes	15	39.5	38	47.5			
**SMA Stranding**	no	29	76.3	46	57.5	0.036	80%	76%
	yes	9	23.7	34	42.5			
**Borderline novel Definition**	BR-	30	78.9	58	72.5	0.505	73%	79%
	BR+	8	21.1	22	27.5			

SMA: superior mesenteric artery; ISGPS: International Study Group of Pancreatic Surgery; IAP: International Association of Pancreatology; SMPV: superior mesenterico-portal vein; PVR: portal vein resection; LNR: lymph node ratio; BR+: Borderline resectable; BR-: Not borderline resectable.

## Supplementary Materials

The following are available online at https://www.mdpi.com/2072-6694/12/4/882/s1, Table S1: STARD 2015 List of Study Items.
