# Supplementary material for: Systematic Analysis of Accuracy in Predicting Complete Oncological Resection in Pancreatic Cancer Patients—Proposal of a New Simplified Borderline Resectability Definition
Source: Cancers (Basel). 2020 Apr 4;12(4):882. doi: 10.3390/cancers12040882 (PMC7226508; doi:10.3390/cancers12040882)
Supplement: Supplementary file 1 [file cancers-12-00882-s001.pdf]

**Supplementary Table S1** Long-term survival estimates

| Long-term survival estimates | | | | | | | | | | |
|---|---|---|---|---|---|---|---|---|---|---|
| | | | Univariate | | | | Multivariate | | | |
| Parameter | median OS | deaths n | HR | 95%CI (lower, | upper) | p-value | HR | 95%CI (lower, | upper) | p-value |
| **Age** | | | | | | | | | | |
| < 68 | 30 | 26 | | | | | | | | |
| > 68 | 22 | 34 | 1.463 | 0.872 | 2.455 | 0.149 | | | | |
| **Sex** | | | | | | | | | | |
| male | 24 | 34 | | | | | | | | |
| female | 26 | 26 | 0.816 | 0.487 | 1.367 | 0.440 | | | | |
| **BMI (kg/m2)** | | | | | | | | | | |
| < 30 | 26 | 50 | | | | | | | | |
| > 30 | 26 | 10 | 0.975 | 0.493 | 1.928 | 0.943 | | | | |
| **ASA Score** | | | | | | | | | | |
| 0-2 | 31 | 27 | | | | | | | | |
| 3-4 | 22 | 33 | 1.706 | 1.014 | 2.869 | **0.044** | 0.628 | 0.269 | 1.466 | 0.282 |
| **Serum CA 19-9** | | | | | | | | | | |
| <40 U/l | 22 | 20 | | | | | | | | |
| >40 U/l | 25 | 30 | 0.946 | 0.410 | 2.179 | 0.895 | | | | |
| **Borderline ISGPS 2014 Definition** | | | | | | | | | | |
| BR- | 30 | 30 | | | | | | | | |
| BR+ | 19 | 30 | 1.771 | 1.056 | 2.969 | **0.030** | 0.649 | 0.290 | 1.453 | 0.293 |
| **Borderline IAP 2017 Definition** | | | | | | | | | | |
| BR- | 40 | 11 | | | | | | | | |
| BR+ | 20 | 49 | 2.808 | 1.443 | 5.465 | **0.002** | 2.993 | 0.895 | 3.007 | 0.075 |
| **Any SMPV alterations** | | | | | | | | | | |
| no | 31 | 29 | | | | | | | | |
| yes | 18 | 31 | 1.895 | 1.122 | 3.200 | **0.017** | 1.178 | 0.448 | 3.100 | 0.740 |
| **SMA stranding** | | | | | | | | | | |
| no | 30 | 34 | | | | | | | | |
| yes | 13 | 26 | 2.618 | 1.520 | 4.510 | **<0.001** | 3.066 | 1.078 | 5.716 | **0.036** |
| **SMA minimal distance** | | | | | | | | | | |
| < 4mm | 24 | 36 | | | | | | | | |
| > 4mm | 29 | 23 | 0.765 | 0.452 | 1.294 | 0.317 | | | | |
| **Borderline novel definition** | | | | | | | | | | |
| BR- | 29 | 42 | | | | | | | | |
| BR+ | 13 | 18 | 2.272 | 1.263 | 4.088 | **0.006** | 0.671 | 0.212 | 2.216 | 0.498 |
| **PVR** | | | | | | | | | | |
| no | 31 | 33 | | | | | | | | |
| yes | 18 | 27 | 1.941 | 1.158 | 3.254 | **0.012** | 1.294 | 0.714 | 2.346 | 0.395 |
| **Multivisceral resection** | | | | | | | | | | |
| no | 27 | 53 | | | | | | | | |

| | | | | | | | | | | |
|---|---|---|---|---|---|---|---|---|---|---|
| yes | 21 | 7 | 1.373 | 0.621 | 3.035 | 0.434 | | | | |
| **T stage** | | | | | | | | | | |
| T1/2 | 28 | 11 | | | | | | | | |
| T3/4 | 25 | 49 | 1.281 | 0.664 | 2.471 | 0.460 | | | | |
| **N stage** | | | | | | | | | | |
| N0 | 33 | 19 | | | | | | | | |
| N+ | 20 | 41 | 2.004 | 1.107 | 3.629 | **0.022** | 1.424 | 0.702 | 2.889 | 0.327 |
| **M** | | | | | | | | | | |
| M0 | 26 | 56 | | | | | | | | |
| M1 | 23 | 4 | 1.164 | 0.420 | 3.228 | 0.771 | | | | |
| **LNR** | | | | | | | | | | |
| < median 0.08 | 28 | 34 | | | | | | | | |
| > median 0.08 | 21 | 26 | 1.229 | 0.724 | 2.087 | 0.446 | | | | |
| **Grading** | | | | | | | | | | |
| G 1/2 | 28 | 44 | | | | | | | | |
| G 3/4 | 23 | 12 | 1.153 | 0.605 | 2.196 | 0.665 | | | | |
| **Lymphovascular invasion** | | | | | | | | | | |
| L0 | 31 | 34 | | | | | | | | |
| L1 | 17 | 26 | 2.291 | 1.340 | 3.915 | **0.002** | 1.322 | 0.705 | 2.479 | 0.384 |
| **Vascular invasion** | | | | | | | | | | |
| V0 | 30 | 40 | | | | | | | | |
| V1 | 14 | 20 | 2.584 | 1.472 | 4.534 | **0.001** | 2.375 | 1.201 | 4.700 | **0.013** |
| **Perineural invasion** | | | | | | | | | | |
| Pn0 | 40 | 8 | | | | | | | | |
| Pn1 | 22 | 51 | 2.398 | 1.127 | 5.101 | **0.023** | 1.259 | 0.547 | 2.896 | 0.588 |
| **UICC/AJCC R status** | | | | | | | | | | |
| R 0 | 30 | 33 | | | | | | | | |
| R + | 18 | 27 | 1.909 | 1.127 | 3.232 | **0.016** | 0.769 | 0.366 | 1.163 | 1.613 |
| **Leeds/Wittekind R status** | | | | | | | | | | |
| R0 wide | 34 | 15 | | | | | | | | |
| R0 narrow/R1 | 21 | 45 | 1.937 | 1.029 | 3.648 | **0.041** | 1.132 | 0.525 | 2.440 | 0.752 |
| **Adjuvant therapy** | | | | | | | | | | |
| no | 23 | 33 | | | | | | | | |
| yes | 30 | 27 | 0.615 | 0.368 | 1.025 | 0.062 | | | | |

*SMA: superior mesenteric artery; BMI: body mass index; ASA score: American Society if anesthesiologists score; CA19-9: carbohydrate antigen 19-9; ISGPS: International Study Group of Pancreatic Surgery; IAP: International Association of Pancreatology; SMPV: superior mesenterico-portal vein; PVR: portal vein resection; LNR: lymph node ratio*
